# Flame-Made La_2_O_3_-Based Nanocomposite CO_2_ Sensors as Perspective Part of GHG Monitoring System

**DOI:** 10.3390/s21217297

**Published:** 2021-11-02

**Authors:** Matvey Andreev, Vadim Platonov, Darya Filatova, Elena Galitskaya, Sergey Polomoshnov, Sergey Generalov, Anastasiya Nikolaeva, Vladimir Amelichev, Oleg Zhdaneev, Valeriy Krivetskiy, Marina Rumyantseva

**Affiliations:** 1 Department of Chemistry, Lomonosov Moscow State University, 119991 Moscow, Russia; andreev@inorg.chem.msu.ru (M.A.); agnes1992@yandex.ru (V.P.); gak1.analyt@gmail.com (D.F.); vkrivetsky@inorg.chem.msu.ru (V.K.); 2 Russian Energy Agency, Ministry of Energy of the Russian Federation, 129085 Moscow, Russia; Galitskaya@rosenergo.gov.ru (E.G.); Zhdaneev@rosenergo.gov.ru (O.Z.); 3 Scientific-Manufacturing Complex Technological Centre, 124498 Moscow, Russia; S.Polomoshnov@tcen.ru (S.P.); s.generalov@tcen.ru (S.G.); A.Nikolaeva@tcen.ru (A.N.); V.Amelichev@tcen.ru (V.A.); 4 A.V. Topchiev Institute of Petrochemical Synthesis, Russian Academy of Sciences, 119991 Moscow, Russia

**Keywords:** greenhouse gases, CO_2_, gas sensor, semiconductor, lanthanum oxide, nanocomposite

## Abstract

Continuous monitoring of greenhouse gases with high spatio-temporal resolution has lately become an urgent task because of tightening environmental restrictions. It may be addressed with an economically efficient solution, based on semiconductor metal oxide gas sensors. In the present work, CO_2_ detection in the relevant concentration range and ambient conditions was successfully effectuated by fine-particulate La_2_O_3_-based materials. Flame spray pyrolysis technique was used for the synthesis of sensitive materials, which were studied with X-ray diffraction (XRD), Fourier-transform infrared spectroscopy (FTIR), diffuse reflectance infrared Fourier transform spectroscopy (DRIFTs) and low temperature nitrogen adsorption coupled with Brunauer–Emmett–Teller (BET) effective surface area calculation methodology. The obtained materials represent a composite of lanthanum oxide, hydroxide and carbonate phases. The positive correlation has been established between the carbonate content in the as prepared materials and their sensor response towards CO_2_. Small dimensional planar MEMS micro-hotplates with low energy consumption were used for gas sensor fabrication through inkjet printing. The sensors showed highly selective CO_2_ detection in the range of 200–6667 ppm in humid air compared with pollutant gases (H_2_ 50 ppm, CH_4_ 100 ppm, NO_2_ 1 ppm, NO 1 ppm, NH_3_ 20 ppm, H_2_S 1 ppm, SO_2_ 1 ppm), typical for the atmospheric air of urbanized and industrial area.

## 1. Introduction

Global climate change is directly correlated with anthropogenic greenhouse gas (GHG) emissions. For hundreds of years, the concentration of CO_2_ in the Earth’s atmosphere did not exceed 300 ppm, but an increase in concentration began to be observed with the beginning of industrialization [1,2]. Today, the CO_2_ concentration is a record 417 ppm, having increased by three ppm in the last year alone. The energy sector contributes the most to climate change and is the source of about 50–60% of anthropogenic GHG emissions [3]. A change in temperature leads to disastrous consequences. Since the 1900s, the average temperature of the Earth’s surface has increased by ~1.07 °C, and the global sea level has risen by ~0.2 m. Anthropogenic CO_2_ emissions are the main reason for the decrease in the ice cover area and ocean acidification. The ocean acidity will reach pH ~7.7 by the end of the century [4,5,6]. To keep the ambient temperature rise within 1.5 °C, according to the Paris Climate Agreement, global anthropogenic CO_2_ emissions should be reduced by about 45% by 2030 compared to the level of 2010 [7,8,9,10,11]. To limit global climate change and mitigate its consequences, it is necessary to achieve net-zero carbon dioxide emissions by 2050. The path to net-zero GHG emissions requires all States to significantly strengthen their energy and climate policies. Thus, for the first time in the world, the EU has legislated a plan to achieve climate neutrality by 2050 with an interim goal of reducing GHG emissions by at least 55% by 2030 [12,13,14,15,16].

**Figure 1:**
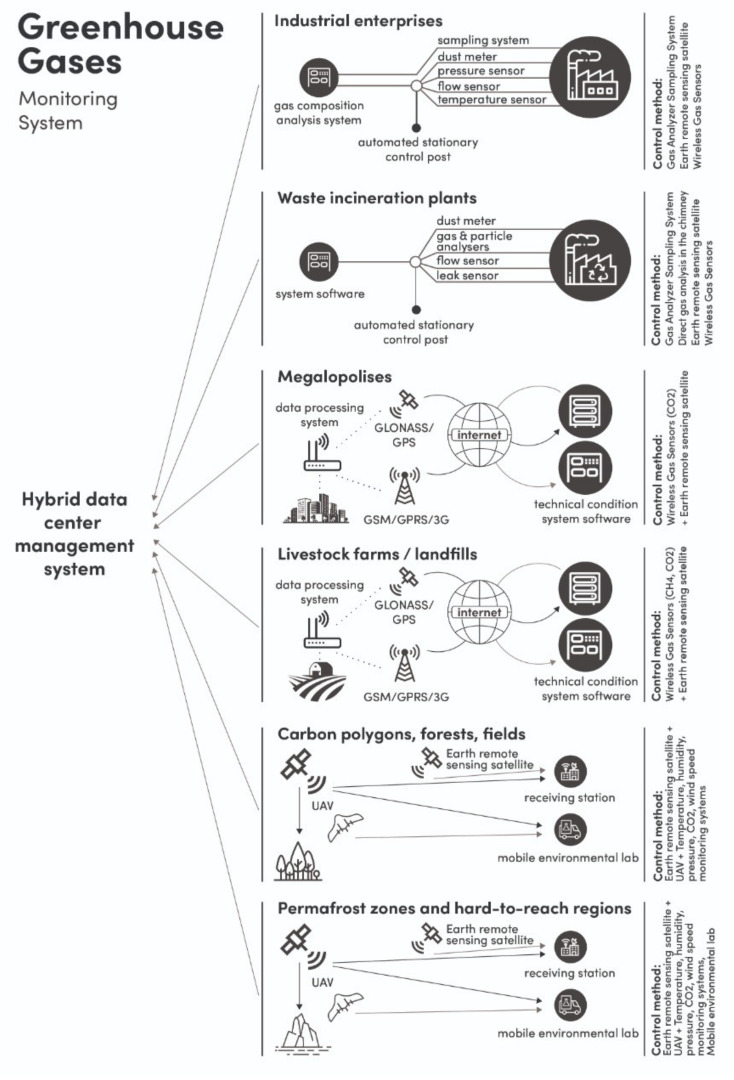
Architecture of the global carbon balance monitoring system.

Achieving carbon neutrality is impossible without a clear accounting and control of emissions of carbon dioxide (CO_2_), methane (CH_4_) and nitrogen oxides (NO_x_) since the emissions of these gases are closely related to the human activity [17,18,19]. Today, the estimation of GHG emissions is mainly carried out by calculation methods with a high degree of uncertainty. The error in accounting for emissions of the main anthropogenic greenhouse gases (CO_2_, CH_4_, NO_x_) is 10–30%, and at the country level can exceed 100%. For the state accounting of GHG emissions, an important task is to create a unified system for monitoring the carbon balance, including accurate determination of emissions from the main GHG emitters, such as industry, housing, transport, agriculture, burning of fossil fuels and waste, etc., and a qualitative assessment of the sequestration potential of ecosystems. The architecture of the global carbon balance monitoring system is shown in Figure 1.

An integrated approach should provide a monitoring system at the global and local levels. On the one hand, remote monitoring of large areas is needed using unmanned aerial vehicles, high-orbit and low-orbit spacecraft equipped with instruments with high spatial and temporal resolutions. On the other hand, precise local control is needed using ground-based detection systems, including stationary gas analysis installations, mobile laboratories and wireless sensor networks. This approach includes measurements at different scales: a country level, a specific region or city, a single enterprise or plant, etc.

Reliable monitoring of the carbon balance of GHG emitters, such as fuel and energy companies, waste incineration plants, metallurgical plants, fertilizer production, etc., should be provided by remote sensing, unmanned aerial vehicles (UAVs) with appropriate equipment, and local ground-based monitoring systems. Ground-based sensors are also used to verify data from remote sensing. The local ground-based monitoring system must be applied to plants and enterprises whose activities are accompanied by GHG emissions, the mass of which is equivalent to 50,000 tons of CO_2_ per year. For monitoring GHG in hard-to-reach areas of the country (permafrost zones, bogs, taiga, etc.) or agricultural sectors, as well as for assessing the sequestration potential of soils and forests, a combination of wireless ground systems and mobile laboratories can be a relevant method. Unmanned aerial vehicles can supplement GHG observations by providing high-resolution vertical profiling, horizontal flow mapping and 3D measurements near the ground. For the global monitoring of GHG balance, remote sensing can be a powerful tool. With remote sensing, it is possible to measure GHG in places where ground-based monitoring is impossible or impracticable. The necessity of using measuring equipment at different heights is explained by the importance of solving the inverse problem; that is, studying the ecosystem sequestration potential. Also, an important task is to link instrumental measurements with a complex atmospheric model for predictive calculations of the plumes movement. The comprehensive methodology should include a strong verification component that collects independent data from different sources. Verification activities can be carried out on multiple spatial and temporal scales and include data from standard computational inventory methods, instrumental methods, remote sensing and modeling. All information must be processed by a hybrid data center system [20,21,22,23,24,25,26,27,28].

Currently, non-dispersive IR spectroscopy (CO_2_), near-IR laser absorption spectroscopy (CH_4_), portable laser analyzers based on CRDS (Cavity ring-down spectroscopy) and ICOS (Integrated cavity-output spectroscopy) (N_2_O) technologies are used to analyze concentrations and fluxes of greenhouse gases (including at meteorological stations and carbon landfills) [29,30,31]. The high cost of optical gas analyzers makes it impossible to deploy widespread networks for monitoring greenhouse emissions and air pollution in real time with high spatial resolution necessary to detect sources of emissions, assess their scale and promptly eliminate them. For this reason, research groups around the world are developing various sensor technologies for monitoring greenhouse gases [28,31,32]. A promising approach is the creation of widespread networks of miniature semiconductor gas sensors. The advantages of such sensors include low cost, high sensitivity, low gas detection limit, a wide range of detectable components, miniature size and low power consumption. Static networks of gas sensors can create a spatially resolved picture of pollution changes in cities, in various landscape systems, territories of industrial enterprises, zones of extraction, processing and transport of fossil fuels. Works are underway to improve the reliability of detection, including through data processing methods [33,34,35,36,37,38,39,40,41].

Carbon dioxide CO_2_ is the most common greenhouse gas of anthropogenic origin. Carbon dioxide is a rather inert substance, and its chemical contact with the semiconductor sensitive material has a weakly expressed chemisorption character. In the last decade, the sensitivity, selectivity, detection limit and response/recovery time of the semiconductor sensors in CO_2_ detection have been significantly improved due to the study of a wide range of materials and the development of new synthesis methods [28,32,38]. Some characteristics of metal oxide semiconductor (MOS) gas sensors are summarized in Table 1 [42,43,44,45,46,47,48,49,50,51,52,53,54,55,56,57,58,59,60,61,62,63,64,65,66,67,68,69]. The highest sensor response is demonstrated by materials based on lanthanum oxide [69]. However, for the widespread use of semiconductor sensors for continuous long-term monitoring of GHG in atmospheric air, it is still necessary: (i) to develop new materials with high selective sensitivity to target gases; (ii) to develop scalable technologies for the synthesis of materials and the formation of a sensitive layer that allows production of a large number of sensor elements with identical characteristics. For these reasons, in this article, sensitive materials based on lanthanum oxide were obtained by flame spray pyrolysis (FSP). The FSP method makes it possible to obtain both ultrafine powders of metal oxides for the subsequent formation of thick-film gas-sensitive elements, as well as directly form highly porous thick films of sensitive materials on the surface of MEMS-microheaters provided with electrode system for electrical measurements.

## 2. Materials and Methods

Gas sensitive materials were synthesized by the flame-spray pyrolysis (FSP) technique with the use of the setup, described previously [70]. La(III) -2-ethylhexanoate (LEH) has been used as precursor. LEH has been synthesized according to the following technique: first La(OH)_3_ was deposited from the La(NO_3_)_3_ aqueous solution by addition of excessive amounts of NH_3_·aq (25% mass). The deposit was centrifugated and washed with distilled water 5 times. The obtained deposit was put into a flask with a Dean-Stark trap with reflux condenser and thermometer. The deposit was added with 10-fold excess of 2-ethylhexanoic acid and heated up to 175 °C under continuous stirring until the end of water accumulation in the trap. The obtained yellowish viscous matter was separated in the separation funnel and studied in order to establish exact La content. The sample was taken in 100 μL volume and treated with 500 μL of concentrated nitric acid. After the sample decomposition, it was diluted by distilled water up to 10 mL of total volume. This solution was further diluted by distilled water 100 times prior to the analysis. La content was determined by total reflection X-ray fluorescence with the S2 PICOFOX instrument (Bruker Nano GmbH, Berlin, Germany). The diluted sample (5 μL) was deposited on the quartz substrate, dried and analyzed. Mo K_α_ radiation was used for X-ray fluorescence ignition. The spectra were collected for 250 s. Internal La standard was used for calculations. The La content in the obtained LEH solution was 50 mg/mL.

The LEH solution was diluted to 0.2 M with toluene before FSP process. This mixture was supplied to the nozzle with 3 mL/min rate and atomized with the oxygen flow at 3 bar pressure drop. Two different oxygen flow rates were used during synthesis, 2 and 1.5 L/min. The powder materials were collected on the glass fiber filters (Whatman GF/A), placed 90 cm above the nozzle with the aid of vacuum pump. The obtained powders were divided in two parts, one of which for each obtained material was annealed at 500 °C in the flow of dry clean air (dry clean air generator GChV 2.0, Himelektronika, Moscow, Russia) for 24 h.

The as prepared materials were studied by X-ray diffraction (XRD) using a DRON-4M diffractometer (Cu Kα, λ = 1.5406). The crystallite size (dXRD) of phases in obtained materials was estimated using the Scherrer formula. The specific surface area was measured by low temperature nitrogen adsorption technique with the use of ChemiSorb 2750 apparatus (Micromeritics, Norcross, GA, USA) and further calculation according to BET model. The morphology of the powders was studied by scanning electron microscopy (SEM) using a Carl Zeiss SUPRA 40 FE-SEM instrument with Inlens SE detector (accelerating voltage 5 kV, aperture 30 μm). The Fourier-transform infrared (FTIR) spectra of samples in the transmittance mode were taken with a Frontier (Perkin Elmer, Walham, MA, USA) spectrometer in transmission mode within the range 400–4000 cm^−1^ with 1 cm^−1^ steps. The 7 mm diameter pellets of the samples were pressed from the 0.5 mg of studied material finely grinded with 50 mg of KBr. In situ diffuse reflectance infrared Fourier transform (DRIFT) spectroscopy was used to investigate the solid-gas interactions. The spectra were registered by a Frontier (Perkin Elmer) spectrometer with the DiffusIR annex and flow chamber HC900 (Pike Technologies, Madison, WI, USA) sealed by a ZnSe window. The DRIFT spectra were registered in the wavenumber range of 800–4000 cm^−1^ with a resolution of 4 cm^−1^ and averaging 30 scans. The powders (30 mg) were placed in alumina crucibles (5 mm diameter). The DRIFT spectra were registered at 410 °C under an exposure to humidified air flow (100 mL/min) containing 400 ppm CO_2_.

**Figure 2:**
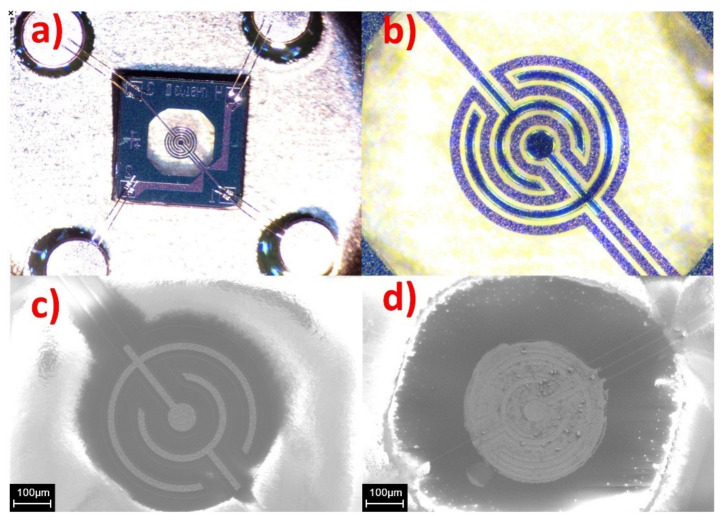
(**a**) Optical image of sensor MEMS crystal, fixed in TO-5 case; (**b**) close optical image of an insulating membrane, bearing a Pt micro-heater, electrically insulated from Pt interdigitated contacts; (**c**) SEM image of heated area with exposed Pt interdigitated contacts; (**d**) SEM image of heated area, uniformly covered by sensitive layer of La-1.5 material.

Sensors were fabricated with the use of micro-hotplates square, 2 × 2 mm MEMS crystals, which were fabricated at the facility of Scientific-Manufacturing Complex “Technological centre” (Zelenograd, Russia). MEMS crystals have thin (~1.5 μm) dielectric membrane (Si_3_N_4_ on top of SiO_2_), which bears thin film Pt micro-heating element and Pt electrodes electrically insulated from each other by additional SiO_2_ layer (Figure 2a–c). The diameter of heated area is 300 μm, which results in 70 mW power consumption at 400 °C. This, along with planar construction, makes this platform perspective from the view of scalability of sensor manufacturing. The crystals were fixed in TO-5 cases (Mars, Torzhok, Russia) using ultrasonic welding (Figure 2a). The obtained materials were deposited on the micro-hotplates in a form of suspensions by inkjet micro-printing technique. The suspension for printing of gas sensitive layers was prepared by ultrasonic treatment in an Elmasonic s15h bath (Elma, Singen, Germany) for 1 h at room temperature. To prepare the suspension, 10 mg of powder were suspended in 1 mL of tetraethylene glycol dimethyl ether. The sensitive layer was deposited using a piezoelectric micro-dispenser NanoTip-HV (Gesim, Radeberg, Germany), which was coupled with lab-made positioning stage, featuring Nippon Bearing (Niigata, Japan) linear actuators and lab made optical microscope system with components, supplied by Altami (St. Petersburg, Russia). The frequency, duration and amplitude of the pulses are 100 Hz, 100 μs and 120 V, respectively. The calculated mass of the applied dry matter was 230 ng (Figure 2d). After deposition, the binder was removed by ramp-heating of the micro-heater in air up to 500 °C. The sensing element was kept at this temperature for 10 h to form a stable thick (2–5 μm) porous film. The morphology of the deposited sensitive layer was investigated by scanning electron microscopy (SEM) with the use of JEOL JSM-6390 LA microscope (JEOL Ltd., Tokyo, Japan). 

The gas sensitivity of the obtained materials in CO_2_ detection was studied in the 200–6700 ppm CO_2_ concentration range. The sensor response towards H_2_ (50 ppm), CH_4_ (100 ppm), CO (20 ppm), NH_3_ (20 ppm), NO (1 ppm), NO_2_ (1 ppm), H_2_S (1 ppm) and SO_2_ (1 ppm) was determined as well in order to investigate the possible cross-sensitivity. The measurements were performed using constant flow air with a pre-assigned concentration of target gas through a gastight polytetrafluoroethylene (PTFE) sensor chamber. Pure air from the pure air generator (GChV 2.0, Himelektronika, Moscow, Russia) was used as a background gas. Due to the unavoidable presence of CO_2_ in generated pure air, in some cases, as mentioned in the text below, synthetic air with 2 ppm CO_2_ was used as background gas. Certified gas mixtures were used as the sources of target gases (Monitoring, St. Petersburg, Russia). Dilution was performed using precision mass gas flow controllers (Bronkhorst, Ruurlo, Netherlands). The measurements were carried out at different humidity conditions, which were pre-assigned and controlled by a P-2 gas flow humidifier (Cellkraft, Stockholm, Sweden). The sensor response *S* = 100% × (*R*_CO2_ − *R_air_*)/*R_air_* was calculated as the difference in electrical resistance of the sensitive layer in the presence of target gas admixture (*R_CO2_*) and in clean air (*R_air_*), related to the resistance in clean air (*R*_air_) and multiplied by 100%.

## 3. Results and Discussion

### 3.1. Materials Morphology and Phase Composition

The obtained materials designations, parameters of synthesis, detected crystalline phases and surface area are summarized in Table 2.

**Figure 3:**
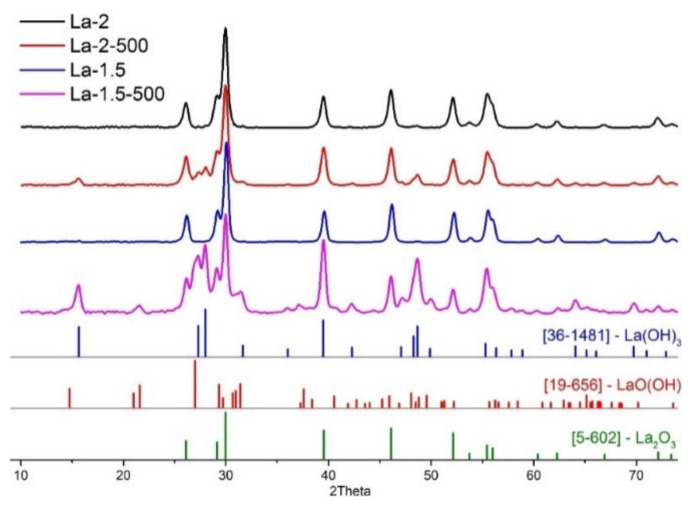
XRD patterns of obtained materials.

**Figure 4:**
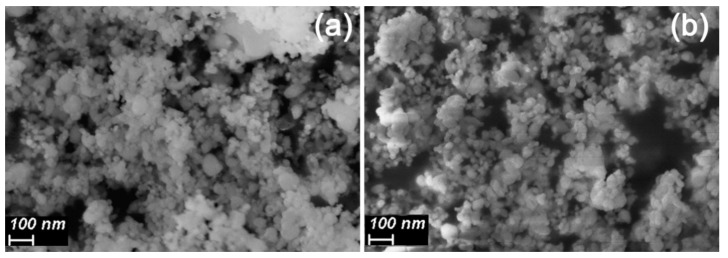
SEM images of La-1.5 (**a**) and La-1.5-500 (**b**) materials.

According to the XRD data, lanthanum oxide is the only crystalline phase, which is present in the as prepared materials after the FSP process (Figure 3). The 24 h annealing in the flow of dry pure air leads to the formation of lanthanum hydroxide. Additionally, in the case of La-1.5-500, some reflections, which may be referred to the formation of lanthanum oxyhydroxide, can be observed. All materials have a quite developed surface, which shrinks to some extent upon annealing in the case of La-2 material and stays the same in the case of material, obtained at the lower oxygen flow during spray formation. The grain size, calculated with the use of Scherrer formula, was 14–16 nm for all detected crystalline phases. SEM images (Figure 4) show that the powders are formed by agglomerates with a size of 30–70 nm, and additional annealing at 500 °C does not lead to a change in the morphology and particle sizes. 

**Figure 5:**
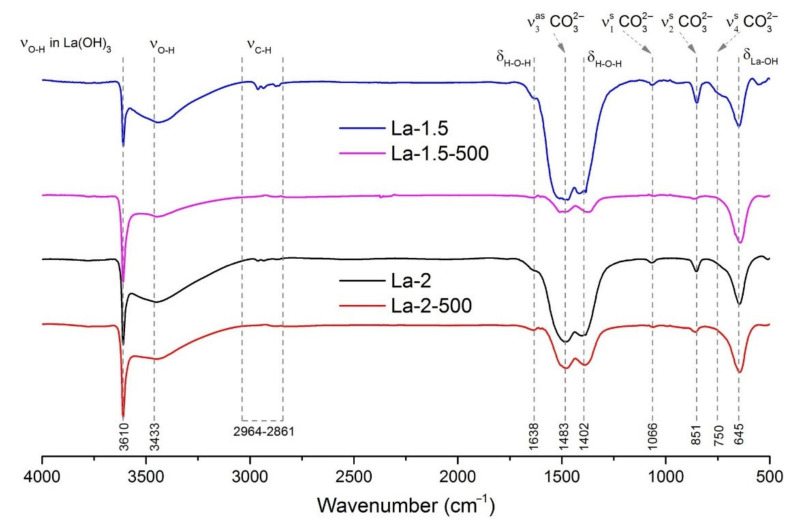
FTIR spectra of obtained materials.

The IR spectroscopy method turns out to be more informative for detecting amorphous phases that do not give a diffraction pattern. FTIR data (Figure 5) indicates the presence of lanthanum hydroxide in both as prepared and annealed materials that is reflected by the prominent bands at 3610 cm^−1^ and 645 cm^−1^, responsible for bulk OH stretching and bending, respectively [71]. The broad band with a maximum at 3433 cm^−1^, as well as the band at 1638 cm^−1^, indicate quite high water content in the obtained materials, which decreases to some extent upon 24 h annealing at 500 °C. Notably, small absorption bands, related to symmetric and asymmetric stretching C-CH_2_ and C-CH_3_ oscillations (2964–2861 cm^−1^), are observed in as prepared samples, indicating some residual hydrocarbon content due to incomplete combustion of organic matter, specifically precursor and fuel components.

The bands at 1483, 1066, 851 and 750 cm^−1^ are related to the carbonate anion vibrations ν_3_, ν_1_, ν_2_ and ν_4_ respectively, while the absorption maximum at ca. 1402 cm^−1^ may be related to the bending vibrational mode of chemisorbed water molecules [72]. The observed spectra reflect the formation of lanthanum carbonate and oxycarbonate phases during FSP process. These absorption bands significantly decrease in intensity after materials annealing at 500 °C. The ratio I_3610_/I_1483_ of vibration intensities at 3610 cm^−1^ (hydroxyl groups) and at 1483 cm^−1^ (carbonate groups) reflects the relative content of carbonate-containing phases in synthesized samples (Table 2). It is obvious that the La-1.5 sample obtained at lower oxygen flow in FSP process is characterized by a higher concentration of carbon-containing phases. Annealing at 500 °C leads to a decrease in the relative content of carbonate and oxycarbonate species. The simultaneous appearance of the La(OH)_3_ and LaOOH phases observed by XRD (Table 2) indicates the process of high-temperature hydrolysis of carbonate particles by water vapor.

### 3.2. Gas Sensor Properties 

**Figure 6:**
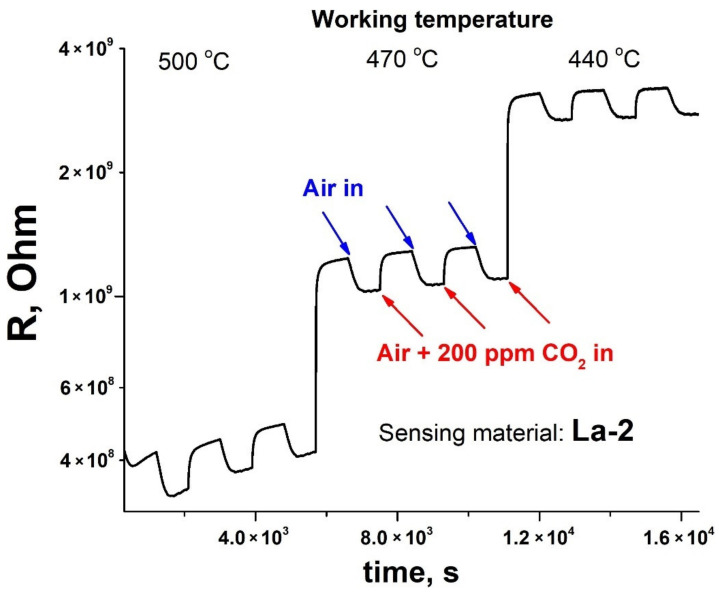
Electrical resistance transient of La-2 material at different working temperatures in the flow of generated dry air and in the flow of air with admixture of 200 ppm CO_2_ above background (a.b.).

In the presence of CO_2_, the resistance of the samples increases and returns to the initial value in pure air (Figure 6) in accordance to previously observed by other researchers [69]. 

**Figure 7:**
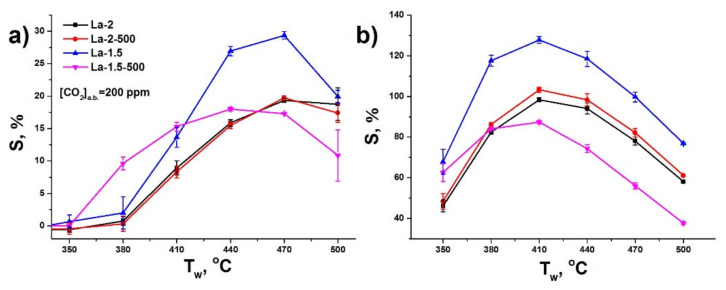
The temperature dependence of sensor response towards 200 ppm CO_2_ above background (a.b.) (**a**) in dry air; (**b**) at 30% relative humidity (at room temperature).

**Figure 8:**
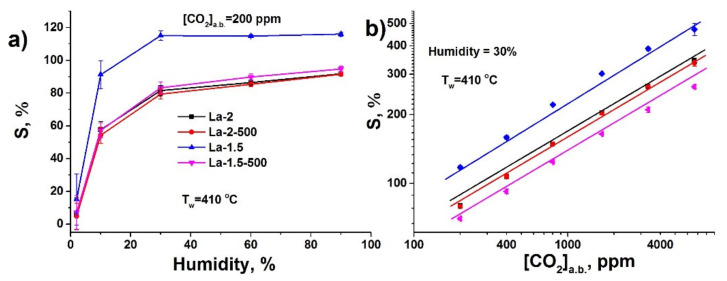
(**a**) Sensor response towards 200 ppm CO_2_ above background (a.b.) in generated air with different relative humidity (working temperature 410 °C); (**b**) Sensor response vs. CO_2_ concentration above background (a.b.) in generated air (relative humidity 30%, working temperature 410 °C).

The temperature dependence of the sensor response towards CO_2_ demonstrates maximum at 470 °C, which is shifted down to 410 °C with the increase in air relative humidity (Figure 7). In can be noted that the response increase with the growth of the ambient humidity is most prominent in the range below 30% RH (Figure 8a). Above this level the further humidity increase is associated with minimal signal rise and in the case of La-1.5 material the response stays nearly constant. The dependence of the sensor response on the CO_2_ concentration is in agreement with power law, typical for semiconductor gas sensitive metal oxides (Figure 8b) [73]. It demonstrates good linearity in double logarithmic coordinates in a quite wide range of concentrations between 200 ppm up to 0.67% above the background level of CO_2_.

**Figure 9:**
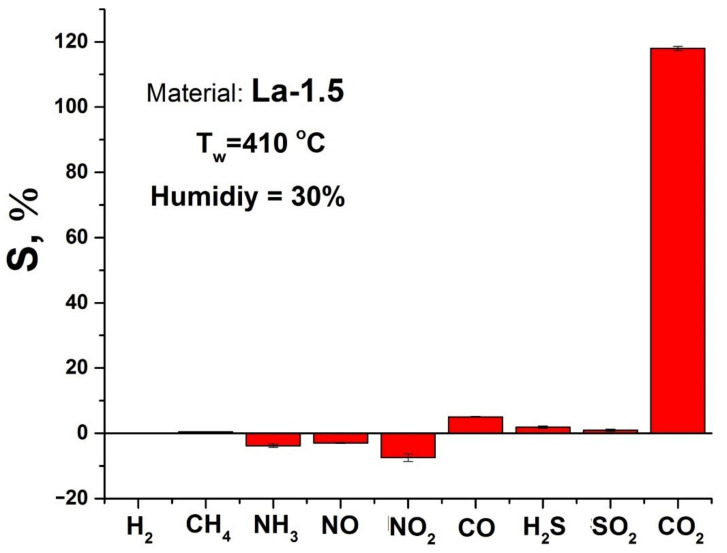
Cross-response of La-1.5 material towards H_2_ (50 ppm), CH_4_ (100 ppm), NH_3_ (20 ppm), NO (1 ppm), NO_2_ (1 ppm), CO (20 ppm), H_2_S (1 ppm) and SO_2_ (1 ppm) in comparison with the response to CO_2_ (200 ppm). Working temperature 410 °C, relative humidity 30%.

Among the obtained materials, the La-1.5 sample demonstrated the best performance, so further studies were focused on it. The cross-sensitivity of this material to the other components in concentrations relevant to atmospheric air monitoring can be considered quite low (Figure 9).

The most prominent cross-response is obtained in the case of nitrogen dioxide, however, it has a negative sign, as this gas possesses oxidative properties. Its adsorption on the materials surface leads to the decrease in free electron density and increase in the *p*-type conductivity. A similar phenomenon is observed in the case of NO. Although, this gas is a reducing one, its interaction with the sensitive layer at elevated temperatures leads to formation of NO_2_ molecules, which is mediated by oxygen on the surface of semiconductor oxide [74].

(1) $$2\mathrm{NO}+O_{2\mathrm{ads}}\to 2{\mathrm{NO}}_{2}$$

Adsorption of this newly formed NO_2_ dominates the formation of sensor response. The negative sensor response, observed in the case of ammonia may also be due to formation of nitrogen dioxide during NH_3_ oxidation on the metal oxide surface.

(2) $${\mathrm{NH}}_{3}+O_{2\mathrm{ads}}\to 2{\mathrm{NO}}_{2}+3H_{2}O$$

The highest positive sensor cross-response is observed in the case of CO, which is obviously due to oxidation of this adsorbed molecule into CO_2_ with the further sensor process.

(3) $$2\mathrm{CO}+O_{2\mathrm{ads}}\to 2{\mathrm{CO}}_{2}$$

**Figure 10:**
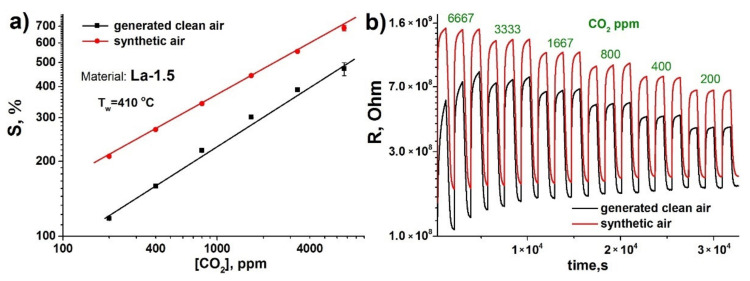
(**a**) Sensor response of La-1.5 material vs. CO_2_ concentration in generated and synthetic air; (**b**) dynamic response of La-1.5 material towards different CO_2_ concentrations in generated and synthetic air. Working temperature 410 °C, relative humidity 30%.

The comparison of the concentration dependence of the sensor signal towards CO_2_, obtained in the generated pure air and synthetic air without CO_2_ background concentrations, reveals almost two-fold improvement of response in the latter case (Figure 10a).

Moreover, the distinctive rise of the baseline resistance of the material in the flow of generated air can be observed (Figure 10b). These phenomena can be attributed to the presence of the unaccounted amounts of nitrogen oxides in the generated air, as the air generator purifies air only from particulate matter, humidity (below 10 ppm at the outlet) and organic compounds (total organic matter content below 0.1 ppm). The adsorption of nitrogen dioxide on the materials surface not only leads to the decrease of the sensitive layer electrical resistance, but also hampers the adsorptive interaction with CO_2_, decreasing the response. Given the observed background response level, the lower CO_2_ detection limit was calculated at 3.5 and 2.5 ppm for generated air and synthetic air, respectively.

**Figure 11:**
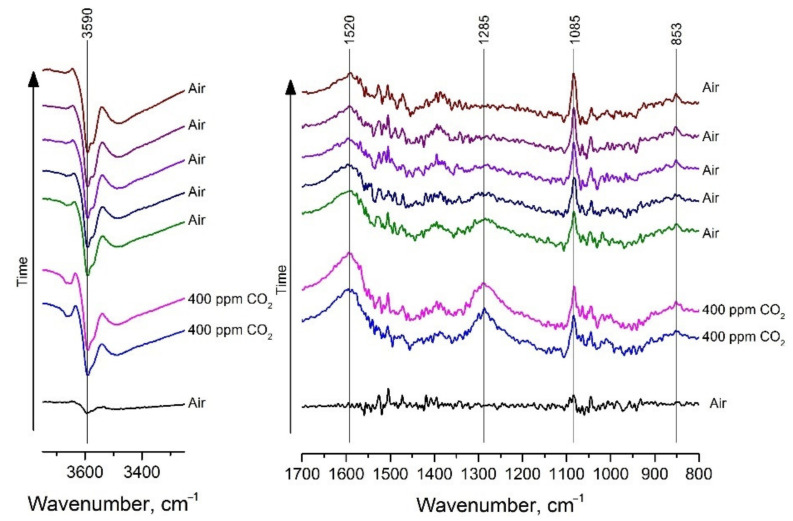
Evolution of DRIFT spectra of La-1.5 material at 410 °C in humidified generated air (RH = 70%) with or without 400 ppm CO_2_.

To clarify the mechanism of the sensor response and to assess the reversibility of the interaction of CO_2_ with the surface of the sensitive material, an operando DRIFT spectroscopy study was effectuated. The experiments were carried out at 410 °C, corresponding to the temperature of sensor measurements, in humidified generated air (RH = 70%) with or without 400 ppm CO_2_. The DRIFT spectra in the range of 3750–3100 cm^−1^, corresponding to the oscillations of hydroxyl groups, and 1700–800 cm^−1^, corresponding to the oscillations of various carbonate species, are shown in Figure 11. In the 3750–3100 cm^−1^ range there is a sharp band at 3590 cm^−1^ corresponding to the oscillations of isolated hydroxyl groups on lanthanum hydroxide surface. The negative intensity of this band indicates a decrease in the concentration of OH groups. This may be due to hydroxyls recombination and desorption at a temperature of 410 °C even in a humid atmosphere. In the 1700–800 cm^−1^ range one can observe the positive bands corresponding to monodentate (1520 and 853 cm^−1^) and bidentate (1285 cm^−1^) surface carbonate species, respectively [69]. A sharp band at 1085 cm^−1^ can be assigned to carbonate group of lanthanum oxycarbonate [71].

**Figure 12:**
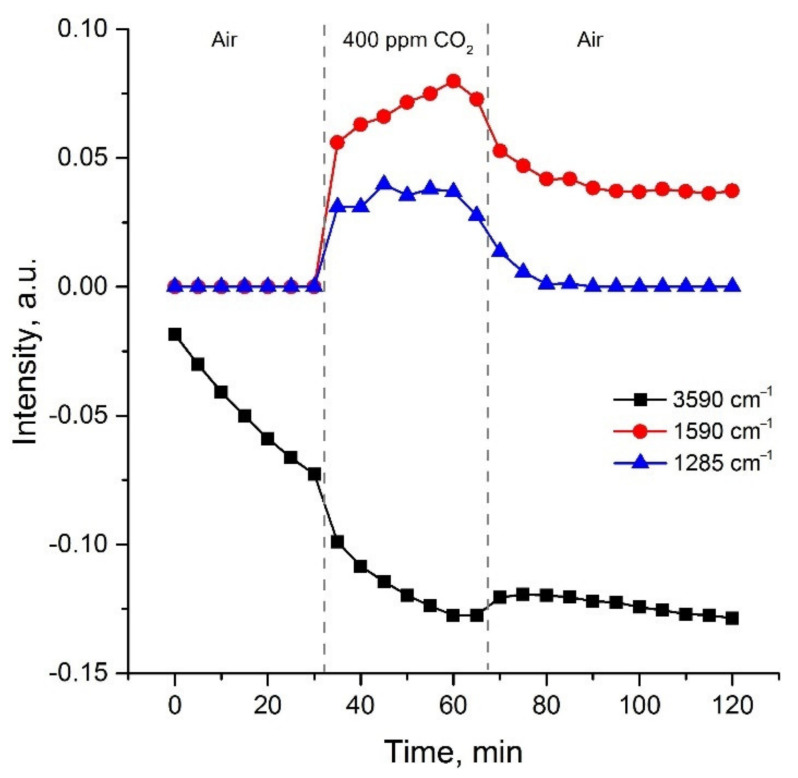
Change in intensities of 3590, 1520 and 1285 cm^−1^ bands depending on exposure time in different gas atmosphere.

Figure 12 demonstrates the change in intensities of 3590, 1520 and 1285 cm^−1^ bands depending on exposure time in different gas atmosphere. We can conclude the hydroxyls concentration is mainly determined by the exposure time at 410 °C but practically does not depend on the presence of CO_2_ in the gas phase. In addition to the main trend, the intensity of OH vibrations decreases slightly with the introduction of CO_2_, and also increases slightly with subsequent exposure in the air. This indicates the replacement of some hydroxyls by adsorbed carbonates due to competitive adsorption [69]. In general, the concentration of hydroxyls approaches a stationary value for two hours at 410 °C. On the contrary, the intensities of carbonate peaks strongly depend on the composition of the gas phase: their intensities increase in the presence of CO_2_, and then decrease with subsequent exposure in the air. The main difference in the behavior of surface bidentate carbonates (band at 1285 cm^−1^) and monodentate carbonates (band at 1590 cm^−1^) is that the former are completely desorbed when CO_2_ is removed from the atmosphere, and the latter are partially preserved on the surface. Such a reorganization of the surface adsorbed groups allows us to expect that preliminary artificial aging in a humid atmosphere with a high concentration of CO_2_ at a temperature of about 400 °C will lead to sufficient long-term stability of sensor characteristics necessary for the sensors integration into GHG monitoring system. 

## 4. Conclusions

Flame spray pyrolysis technique allows to obtain fine particulate composite material with a dominating La_2_O_3_ crystalline phase, accompanied with amorphous La(OH)_3_ and carbonates. Increase in the carbonate phase content, which can be achieved through oxygen-deficit synthesis conditions, leads to the increase in the composite response towards CO_2_. Combination of low CO_2_ detection limit with sensor response independence from air humidity at RH > 30% observed for the obtained La_2_O_3_-based nanocomposite makes it suitable for detecting CO_2_ in atmospheric air at the current concentration level (400 ppm) and below. Semiconductor sensors, based on low power consuming and cost-effective MEMS micro-hotplates with a sensitive layer of synthesized La_2_O_3_-based nanocomposite, are promising elements for creating distributed networks with high spatial and temporal resolution for the GHG monitoring system.

## Figures and Tables

**Table 1:** Summary of literature data on sensor response *S* = 100%×|*R_CO2_* − *R_air_|*/*R_air_* of MOS gas sensors in CO_2_ detection.

Material	Synthesis Method	C (CO_2_), ppm	Operating Temperature, °C	Response ^1^	Ref.
BiOCl-Au	Surfactant assisted	400	300	63	[42]
ZnO-SnO_2_	Spray pyrolysis	500	300	90	[43]
LaOCl-SnO_2_	Electrospinning	1000	300	270	[44]
La_2_O_3_-ZnO	Hydrothermal	5000	400	65	[45]
ZnO	Spray pyrolysis	400	350	64	[46]
CdO-CeO_2_	Co-precipitation	800	250	45	[47]
WO_3_-ZnO	Mechanochemical	1000	450	65	[48]
La_2_O_2_CO_3_	Hydrothermal	5000	300	62	[49]
ZnO-SnO_2_	Hydrothermal	1000	150	350	[50]
La_2_O_3_-SnO_2_-Au	Electrospinning	100	300	10	[51]
SnO_2_	Co-precipitation	2000	240	30	[52]
SnO_2_	Mechanochemical	1000	400	10	[53]
La_2_O_3_	Chemical bath	350	250	55	[54]
LaOCl	Sol-gel	2000	260	240	[55]
NdO_2_CO_3_	Sol-gel	1000	350	300	[56]
La_2_O_3_CO_3_	Co-precipitation	3000	325	600	[57]
LaFeO_3_	Sol-gel	2000	300	120	[58]
LaCaFeO_3_	Sol-gel	1000	320	70	[59]
LaOCl-SnO_2_	Electrostatic spray pyrolysis	2000	425	40	[60]
CuO-BaTiO_3_	Magnetron sputtering	1000	250	80	[61]
LaFeO_3_-SnO_2_	Mixing	4000	250	170	[62]
ZnO-CuO	Mixing	4000	300	30	[63]
SnO_2_-LaOCl	Impregnation	2000	350	150	[64]
SnO_2_-LaOCl	Drop-coating	4000	400	580	[65]
ZnO-LaOCl	Drop-coating	2000	400	250	[66]
SnO_2_-La_2_O_3_	Impregnation	500	250	30	[67]
La_2_O_3_-Pd	Dipping	500	250	40	[68]
La_2_O_2_CO_3_	Thermal decomposition	500	300	700	[69]

^1^ For correct comparison all the data were recalculated as *S* = 100%×|*R_CO2_* − *R_air_|*/*R_air_*.

**Table 2:** Obtained materials and their characteristics.

Material	O_2_ Flow, L/min	Post-Synthetic Annealing	S_surf_, m^2^/g	Phase Composition, XRD	I_3610_/I_1483_, FTIR
La-2	2	--	42	La_2_O_3_	1.05
La-2-500	2	500 °C, 24 h	31	La_2_O_3_, La(OH)_3_	2.12
La-1.5	1.5	--	27	La_2_O_3_	0.56
La-1.5-500	1.5	500 °C, 24 h	27	La_2_O_3_, La(OH)_3_, LaO(OH)	5.12

## Data Availability

Not applicable.
